# Construction and validation of a nomogram for identifying the patients at risk for rapid progression of advanced hormone-sensitive prostate cancer

**DOI:** 10.1186/s12885-025-14035-w

**Published:** 2025-04-08

**Authors:** Xiaolong Xu, Weiyu Fei, Mingshuang Wu, Yi He, Bo Yang, Cuicui Lv

**Affiliations:** 1 Department of Urology, The Second Affiliated Hospital of Dalian Medical University, Dalian, Liaoning China; 2 Department of Emergency Intensive Care Unit, The Second Affiliated Hospital of Dalian Medical University, Dalian, Liaoning China; 3 Department of Endocrine, The Second Affiliated Hospital of Dalian Medical University, Dalian, Liaoning China

**Keywords:** Hormone-sensitive prostate cancer, Fasting triglyceride-glucose, Lactate dehydrogenase, Nomogram

## Abstract

**Background:**

This study aimed to evaluate the prognostic significance of lactate dehydrogenase (LDH) and fasting triglyceride-glucose (TyG) index in advanced hormone-sensitive prostate cancer (HSPC) patients, with the ultimate goal of developing and validating a nomogram for predicting castration-resistant prostate cancer (CRPC) free survival.

**Materials and methods:**

The follow-up data of 207 CRPC patients who had androgen deprivation therapy as their initial and only treatment before progression were retrospectively reviewed. To assess prognostic variables, univariate and multivariate Cox regression analyses were performed. The concordance index (C-index), calibration curves, receiver operating characteristic (ROC) curves, and decision curve analyses (DCA) were utilized to construct and test a novel nomogram model.

**Results:**

TyG index, LDH, M stage and Gleason sum were determined to be independent prognostic markers and were combined to create a nomogram. This nomogram worked well in the tailored prediction of CRPC development at the sixth, twelve, eighteen, and twenty-fourth months. The C-indexes for the training and validation sets were 0.798 and 0.790, respectively. The ROC curves, calibration plots, and DCA all indicated good discrimination and prediction performance. Furthermore, the nomogram had a higher prognostic ability than the M stage and the Gleason sum. The nomogram-related risk score classified the patient population into two groups with significant progression differences.

**Conclusions:**

The created nomogram could help identify patients at high risk for rapid progression of advanced HSPC, allowing for the formulation of tailored therapy regimens and follow-up methods in a timely manner.

## Introduction

Prostate cancer (PCa) remains a leading cause of cancer-related mortality among men, responsible for approximately 390,000 deaths annually worldwide [1]. Since the introduction of PSA screening in China, the incidence of both localized and advanced PCa has increased, with a corresponding rise in cancer-related mortality observed since 2012 [2]. Androgen deprivation therapy (ADT), the cornerstone treatment for locally advanced or metastatic hormone-sensitive prostate cancer (HSPC), inhibits PCa progression by suppressing circulating testosterone levels. Nevertheless, most patients receiving ADT develop castration-resistant prostate cancer (CRPC) within three years [3], with a median survival of only 14 months after CRPC diagnosis [4]. The heterogeneous nature of PCa results in a broad disease spectrum, ranging from clinically indolent to highly aggressive subtypes. According to Hussain et al.‘s hypothesis, patients who progress to CRPC within the first seven months of ADT face a fourfold increased risk of mortality [5]. Large-scale phase III randomized controlled trials, including CHAARTED and LATITUDE [6, 7], have demonstrated that identifying HSPC patients at risk of rapid versus delayed progression to CRPC prior to treatment initiation enables the implementation of tailored follow-up strategies and optimization of therapeutic regimens. Therefore, identifying predictive indicators of CRPC progression in both locally advanced and metastatic PCa is crucial for establishing early monitoring protocols, facilitating timely detection of disease progression, and optimizing treatment strategies, including the judicious use of chemotherapy.

Several clinicopathological features, including age, PSA levels, Gleason score, tumor stage, lymph node involvement, and metastatic status, provide valuable prognostic information for monitoring PCa progression. However, the predictive utility of these conventional markers is often limited, particularly in patients with ambiguous clinical presentations or intermediate-grade/stage disease.

Previous studies have demonstrated a significant association between the triglyceride-glucose (TyG) index—a composite measure of fasting blood glucose and fasting triglycerides—and prognosis across various cancer types [8, 9]. Additionally, lactate dehydrogenase (LDH), a key enzyme in anaerobic glycolysis, has been implicated in tumorigenesis and is essential for sustaining tumor growth [10]. These cost-effective and easily accessible biomarkers are primarily used to assess carcinogenesis risk and predict outcomes in multiple malignancies [11, 12]. However, their roles in the progression of HSPC remain underexplored.

In this study, we retrospectively analyzed the TyG index and serum LDH levels in patients with hormone-sensitive prostate cancer (HSPC) and evaluated their prognostic significance in conjunction with established clinical parameters. Furthermore, we developed a nomogram model to predict disease progression in HSPC patients.

## Methods and materials

### Patient recruitment and clinicopathological variables collection

This retrospective analysis included 207 patients with complete follow-up data between November 1, 2016, and July 1, 2023. The Dalian Medical University Second Hospital’s ethical committee approved the protocol in accordance with the Declaration of Helsinki (certificate number: 2023064). Before any data was collected, all patients provided written informed consent and gave their approval to the medical care, including the use of their medical records for research. During or after data collection, the authors had access to information that may be used to identify specific individuals. All information was anonymised prior to researchers’ access. factors related to clinicopathology at the time of PCa diagnosis, including age, PSA levels, clinical TNM stage, Gleason sum, fasting blood glucose (FBG), triglyceridesis (TG), LDH, and progression-free survival (PFS), which is identified from the date of therapy to the date of CRPC advancement, were collected and given in Table 1. All individuals received subcutaneous injections of LHRH agonists (Goserelin 3.6 mg specifications: once every 28 days, one dosage each time; Leuprorelin 3.75 mg specifications: once every 28 days, one dose each time) at different places on the upper arm, abdomen, and buttocks, combined with anti-androgen (bicalutamide 50 mg orally once day or flutamide 250 mg orally 3 times daily) as primary and only treatment before advancement. Patients who advanced to CPRC fulfilled the following requirements: Patients who met the following criteria were eligible to advance to CPRC: (1) PSA progression: PSA level above 2.0 ng/mL, interval 1 week, three times greater than the baseline level > 50%; (2) Radiological progression: the appearance of new lesions—either two or more new bone lesions on bone scan or a soft tissue lesion using the Response Evaluation Criteria in Solid Tumours. (3) serum testosterone level below 50 ng/dl (1.7 nmol/L). All patients were followed up to the advancement of CRPC.

### Establish a nomogram for predicting the CRPC progression

In this study, 207 patients were randomly divided into a training cohort (*n* = 147, ~ 70%) and an internal validation cohort (*n* = 60, ~ 30%) using the R package ‘caret’. The R package ‘survival’ was employed to integrate data on survival time, survival status, and clinicopathological characteristics. Multivariate Cox regression analysis was performed in the training cohort to evaluate the prognostic significance of these variables. Subsequently, the ‘RMS’ package was utilized to construct nomograms predicting disease progression rates at 6, 12, 18, and 24 months, based on the results of the multivariate Cox proportional hazards analysis. The nomogram provides graphical data for these characteristics, and the prognosis risk of an individual patient can be calculated using the points associated with each risk factor. During nomogram validation, each patient in the validation cohort’s total points were calculated using the established nomogram. The clinical value of the nomogram was assessed using the concordance index (C-index), calibration curves, receiver operating characteristic (ROC) curves, and decision curve analyses (DCAs).

### Analyzed the survival patients with high-risk or low-risk score based on nomogram

The optimal risk score cut-off value for the full dataset was calculated using the R software tool maxstat (maximally selected rank statistics with various p-value approximations, version: 0.7–25). The minimum and maximum sample sizes for each category were set at greater than 25% and less than 75%, respectively. Based on the available information, patients were divided into high-risk and low-risk groups. The Survfit function of the R software program was used to explore the prognosis disparity between the two groups, and the significance of the prognostic difference between the various sample groups was determined using the log-rank test.

### Statistical analysis

The appropriate cutoff values for age, TG, FBG, and TyG were calculated using X-tile software v3.6.1, and all statistical analyses were carried out with SPSS version 24.0. For categorical variables, frequencies and proportions are given. Multivariate Cox regression analysis was used to generate the relevant hazard ratios (HRs) and 95% confidence intervals (CIs). For DCA, the “ggDCA” R package was used. The area under the curve (AUC) was calculated using ROC analysis and the R software package pROC (version 1.17.0.1). We particularly gathered the patients’ follow-up duration and risk score, and then performed ROC analysis using the pROC function at 6, 12, 18, and 24 months. Statistical significance was determined for all analyses with a P value < 0.05.

## Results

### Baseline clinicopathological characteristics

A total of 207 patients who fulfilled the criteria for survival information were enrolled and divided into two cohorts: training (*n* = 147) and validating (*n* = 60). Overall, the median age at diagnosis was 72 years (52–92). At the time of being diagnosed, 56 (27.05%), 103 (49.76%), and 48 (23.19%) patients were classified as M1c, M1a/b, and M0, respectively. 118 patients, or 56.01%, had metastases to lymph nodes. T2, T3, and T4 stage diagnoses were made for 40 (19.32%), 65 (31.40%), and 102 (47.28%) patients, respectively. 33 (15.94%), 71 (34.30%), 79 (38.16%), and 24 (11.60%) got scores of 7, 8, 9, and 10, among the Gleason total values. PFS varied from 2 to 71 months, with 16 months serving as the median. Based on the ideal cut-off values, we separated the continuous normal distribution variables—such as age, FBG, TG, TyG, and LDH—into categorical variables. Table 1 provided a summary of the cohorts’ specific features.

**Table 1:** Representativeness of study participants

Clinical characteristics		Number
Age	Median [min-max]	72 [52, 92]
	≤ 63	29 (14.01%)
	> 63	178 (85.99%)
T stage	T2	40 (19.32%)
	T3	65(31.40%)
	T4	102 (49.28%)
N stage	N0	89 (43.99%)
	N1	118 (56.01%)
M stage	M0	48 (23.19%)
	M1a/b	103 (49.76%)
	M1c	56 (27.05%)
Gleason sum	7	33 (15.94%)
	8	71 (34.30%)
	9	79 (38.16%)
	10	24 (11.60%)
t-PSA (ng/dl)	≤ 100	81 (39.13%)
	> 100	126 (60.87%)
TG(mg/dL)	Median [min-max]	95.69 [48.73–396.04]
	≤ 78.00	58 (28.02%)
	> 78.00	149 (71.98%)
FBG(mg/dL)	Median [min-max]	97.92 [66.54–267.84]
	≤ 101.00	122 (58.94%)
	> 101.00	85 (41.06%)
TyG	Median [min-max]	8.48 [7.64–10.18]
	≤ 8.32	74 (35.75%)
	> 8.32	133 (64.25%)
LDH(U/L)	Median [min-max]	210.87 [75.00–994.64]
	≤ 266.00	156 (75.36%)
	> 266.00	51 (24.64%)
PFS (months)	Median [min-max]	16 [2–71]

Abbreviations: TG: triglycerides; FBG: fasting blood glucose; TyG: Triglyceride-glucose index; LDH: lactate dehydrogenase; PFS: progression free survival

### Construction of a combined nomogram for individualized prediction

For univariate and multivariate Cox regression analysis, the clinico-pathologic factors—age, PSA levels, clinical TNM stage, Gleason sum, FBG, TG, TyG, and LDH—were employed (Table 2). According to Table 2, the findings showed that TyG, LDH, M stage, and Gleason sum were independent risk factors for the advancement of HSPC.

**Fig. 1:**
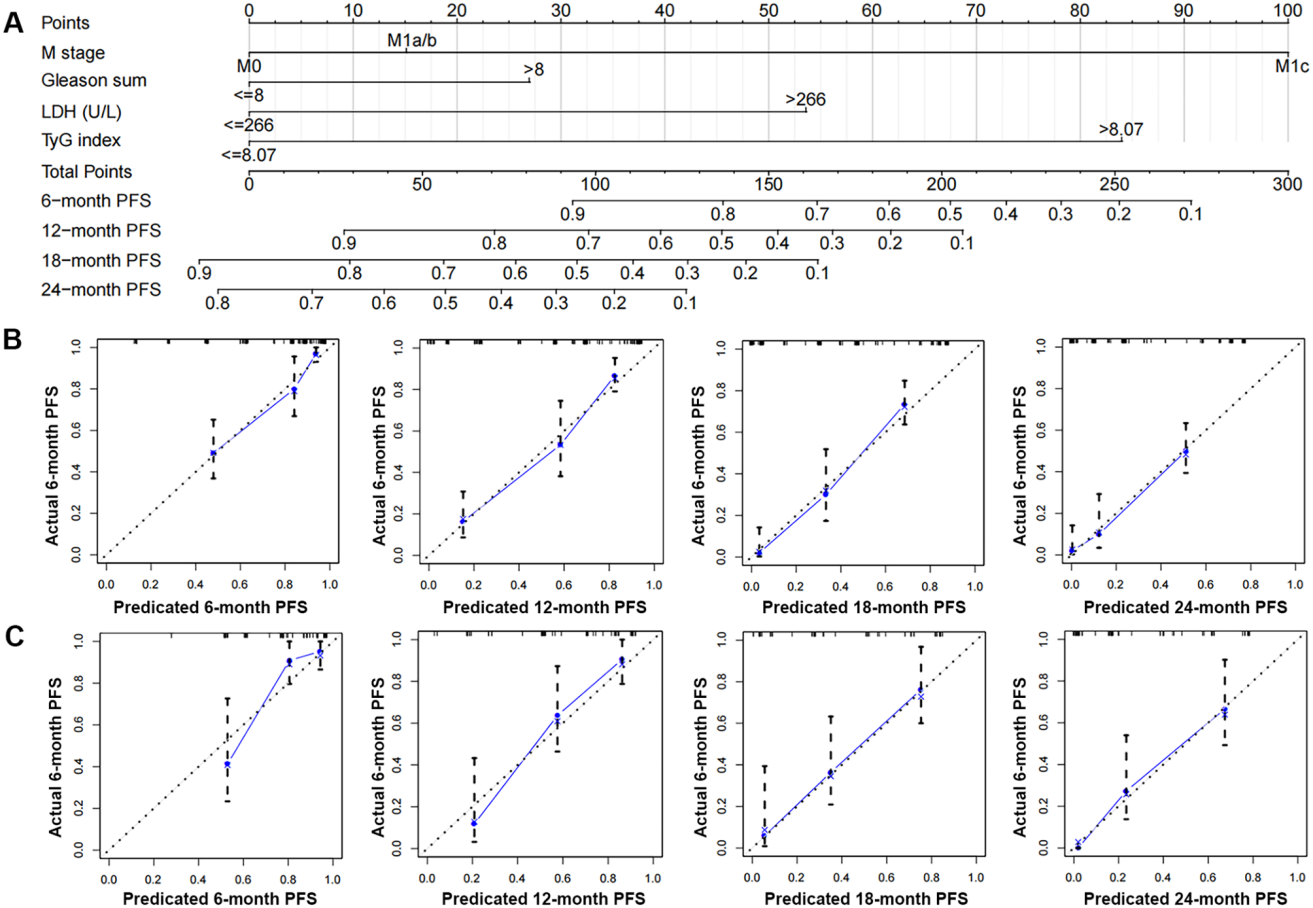
Establishment and validation of a combined nomogram. Nomogram based on fasting triglyceride-glucose (TyG) index and lactate dehydrogenase (LDH), M stage and Gleason sum was constructed to predict the 6-, 12-, 18- and 24-month CRPC-free survival (**A**). Predictive accuracy of the nomogram was assessed by the calibration plots in training cohort (**B**) and validating cohort (**C**)

**Fig. 2:**
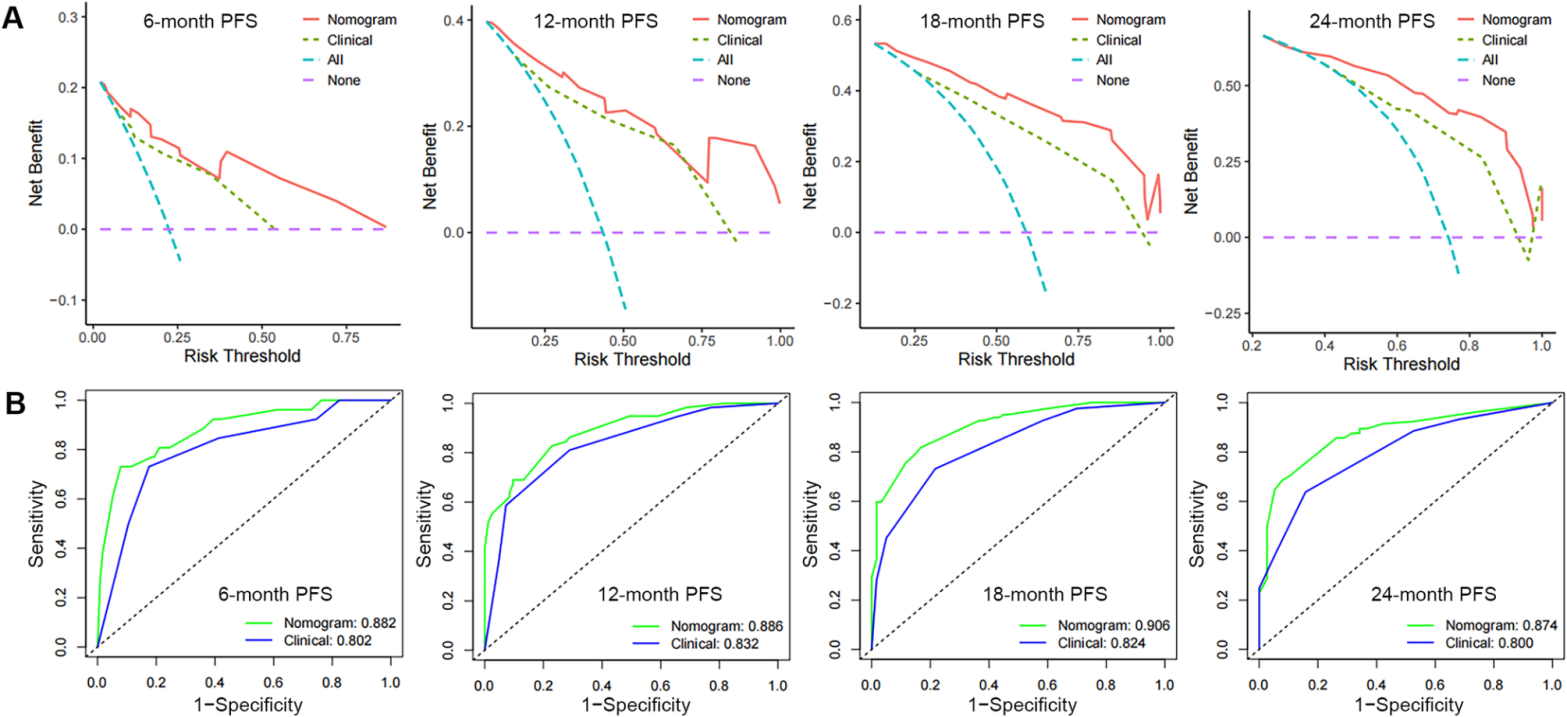
Validation of predictive capacity of the Nomogram in the training cohort. (**A**) Comparing the time-dependent decision curve analysis for the clinical benefit of the nomogram and clinical model; (**B**) Comparing ROC curves of the nomogram and clinical model for 6-, 12-, 18- and 24-month CRPC-free survival

In the training cohort, a nomogram integrated with the TyG, LDH, M stage, and Gleason total was established by multivariate Cox regression analysis (Fig. 1). In both the training and validation cohorts, the calibration plots demonstrated that the nomogram performed well in the customized prediction of progression to CRPC (Fig. 1B, C). We created a clinical model based on the Gleason sum and M stage to demonstrate the incremental usefulness of the TyG and LDH in predicting outcomes. The nomogram’s C-index in the training set was higher than the clinical model’s (0.719, 95% CI: 0.675–0.762), indicating good consistency. It was 0.798 (95% CI: 0.762–0.833). Within the real threshold probability range, the decision curves demonstrated that the nomograms’ clinical efficacy outperforms the clinical model’s (Fig. 2A). The AUC values of the nomogram for predicting the PFS were all greater than those of the clinical model, according to the ROC analysis in the training set (Fig. 2B).

**Table 2:** Univariate and multivariate analyses of factors associated with HSPC progression

Variables		Univariate analysis		Multivariate analysis	
		HR (95%CI)	*P* value	HR (95%CI)	*P* value
Age	≤ 63	-	-	-	-
	> 63	0.51 (0.32–0.84)	0.008**	0.68 (0.41–1.14)	0.145
Gleason sum	≤ 8	-	-	-	-
	> 8	2.00 (1.42–2.82) 2.78)	< 0.001***	1.58 (1.09–2.28)	0.014*
T-stage	T2-T3	-	-	-	-
	T4	1.29 (0.93–1.79)	0.133	-	-
N-stage	N0	-	-	-	-
	N1	1.56 (1.13–2.19)	0.007**	1.18 (0.83–1.68)	0.346
M-stage	M0	-	-	-	-
	M1a/b	1.99 (1.28–3.09)	0.002**	1.31 (0.82–2.10)	0.245
	M1c	6.92 (4.12–11.62)	< 0.001***	5.71 (3.18–10.24)	< 0.001***
t-PSA	≤ 100	-	-	-	
	> 100	1.40 (1.01–1.96)	0.045*	1.38 (0.91–1.87)	0.056
TG (g/L)	< 2.25	-	-	-	-
	≥ 2.25	2.54 (1.72–3.75)	< 0.001***	1.04 (0.61–1.76)	0.871
FBG(mg/dL)	≤ 101.00	-	-	-	-
	> 101.00	1.61 (1.15–2.26)	0.006**	0.88 (0.60–1.28)	0.516
TyG	≤ 8.32	-	-	-	-
	> 8.32	4.0 (2.68–6.06)	< 0.001***	4.52 (2.63–7.77)	< 0.001***
LDH(U/L)	≤ 266.00	-	-	-	-
	> 266.00	2.61 (1.75–3.85)	< 0.001***	2.04 (1.21–3.46)	0.007**

Abbreviations: TG: triglycerides; FBG: fasting blood glucose; TyG: Triglyceride-glucose index; LDH: lactate dehydrogenase; *P < 0.05, **P < 0.01, ***P < 0.001

### Predictive accuracy validation of the nomogram

**Fig. 3:**
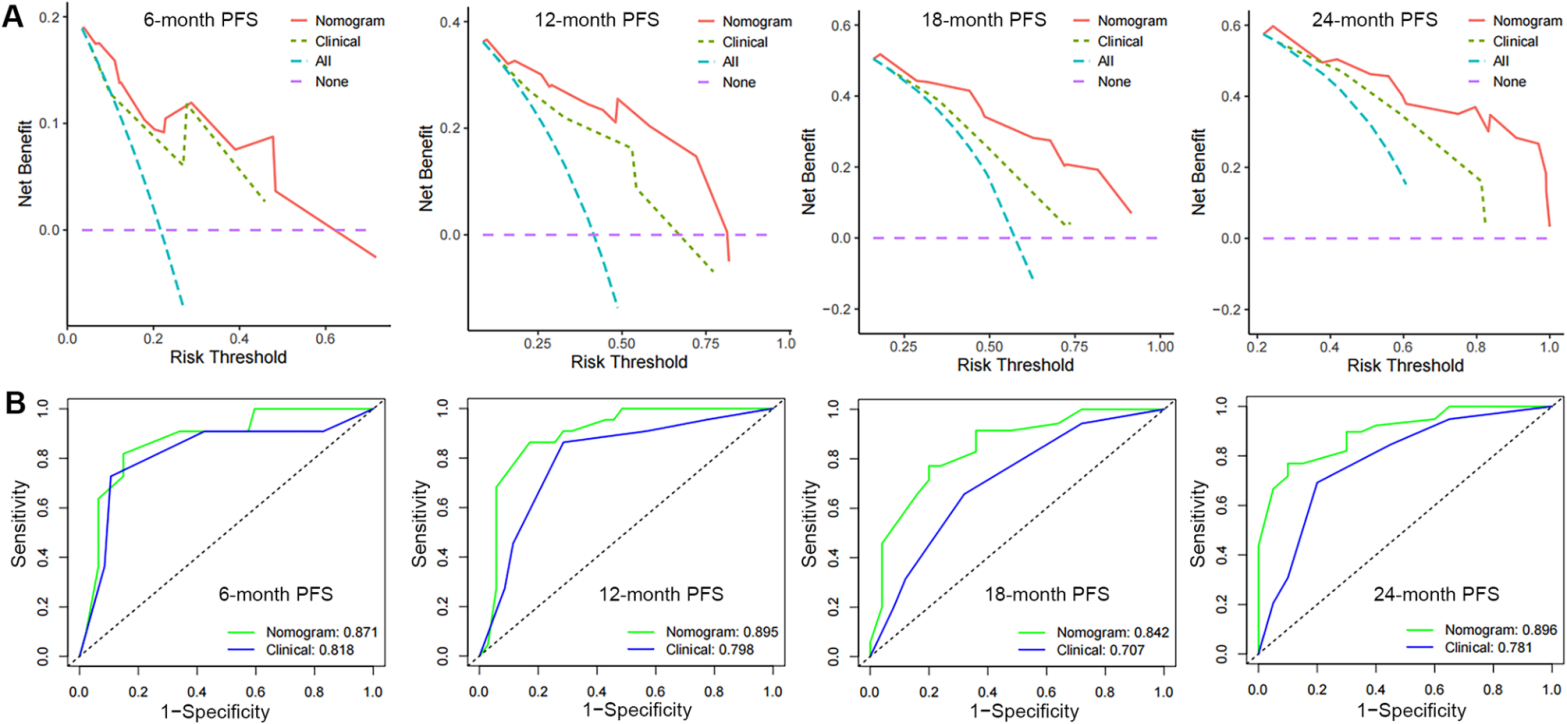
Validation of predictive capacity of the Nomogram in the testing cohort. (**A**) Comparing the time-dependent decision curve analysis for the clinical benefit of the nomogram and clinical model; (**B**) Comparing ROC curves of the nomogram and clinical model for 6-, 12-, 18- and 24-month CRPC-free survival

**Fig. 4:**
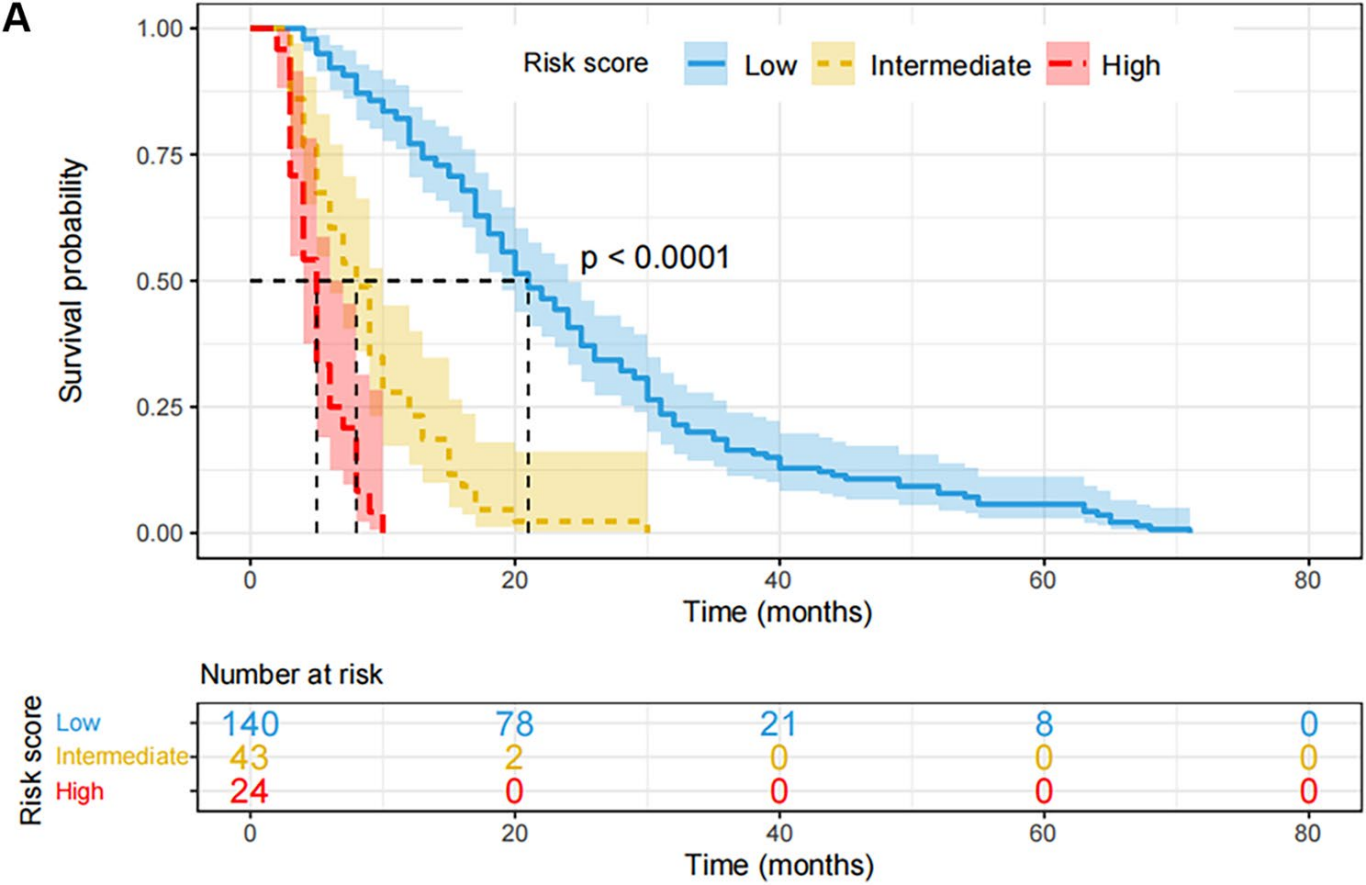
Validation of predictive value of the Nomogram. The progression free survival curves based on nomogram correlated risk score in the whole cohort

The nomogram’s C-index for predicting the progression of CRPC in the validation cohort was 0.790 (95% CI: 0.732–0.845), higher than the clinical model’s C-index of 0.723 (95% CI: 0.644–0.801). Furthermore, the DCA curve showed that, at the 6th, 12th, 18th, and 24th month, the nomogram clearly benefited the none or all strategy, outperforming the clinical model within the real threshold probability range (Fig. 3A). The ROC curve indicates that the nomogram’s 6th, 12th, 18th, and 24th AUCs were, respectively, 0.871, 0.895, 0.842, and 0.896 (Figs. 3B). These values were all higher than the AUCs (0.818, 0.798, 0.707, and 0.781, respectively) that the clinical model anticipated. All of the participants in this study were combined into a single risk score using nomogram modeling. In addition, the optimal cutoff value for the risk score was determined. Patients were divided into high, intermediate, and low risk groups based on this information; 11.6% (*n* = 24) were in the high risk group, 20.8% (*n* = 43) in the intermediate group, and 67.6% (*n* = 140) were in the low risk group. The PFS of patients in the low-risk group (median: 21 months) was considerably superior than that of the intermediate-risk group (median: 8 months) and the high-risk group (median: 5 months, *P* < 0.0001), according to the K-M survival curves (Fig. 4).

## Discussion

This study examined the connection between the TyG index, LDH, and clinical outcome in patients with advanced HSPC who were treated with LHRH agonists in conjunction with anti-androgen as the primary therapy prior to progression. Additionally, we developed a nomogram model with the TyG index, LDH, M stage, and Gleason sum through multivariate cox analysis. We hypothesized that this model’s predictive significance would assist clinicians in promptly identifying high-risk HSPC patients and in offering appropriate treatment options.

The clinical staging of newly diagnosed HSPC patients in China differs significantly from that in Western countries, with fewer cases presenting as clinically localized disease and a majority diagnosed at locally advanced or metastatic stages. Additionally, the inherent heterogeneity of HSPC results in a broad disease spectrum, ranging from clinically indolent to highly aggressive subtypes. Large-scale randomized controlled phase III trials, such as CHAARTED [6] and LATITUDE [7], have demonstrated that patients with high-volume or high-risk HSPC achieve significantly improved overall survival when treated with ADT combined with docetaxel or abiraterone acetate during the early hormone-sensitive phase. Consequently, the ability to predict and identify HSPC patients at high risk of rapid CRPC progression is of critical importance for optimizing treatment strategies.

Using multiple regression analysis, we newly concluded that TyG index and LDH were independent risk factors for rapid progression of HSPC patients. After being first proposed as a diagnostic of insulin resistance (IR), the TyG index—which is derived from fasting blood glucose and triglycerides—has been linked to cancer [13]. While the precise mode of action of the TyG index on cancer remains unclear, a number of plausible explanations have been suggested. First off, it has been suggested that IR syndrome plays a significant role in cell proliferation [14]. According to pertinent research, hyperinsulinemia may impact energy metabolism by increasing the uptake of glucose by cells. This, in turn, may activate specific signal transduction pathways in cells, which in turn may stimulate cell proliferation and inhibit cell apoptosis, ultimately contributing to the development of cancer [15]. Furthermore, insulin’s structurally related IGF-1 receptor binding and activation likely contribute to malignant transformation, cancer development, and cell metastasis [16]. Additionally, elevated levels of blood glucose themselves make cells more susceptible to IGF-1, which encourages the onset and progression of cancer [17]. Lastly, IR starts the inflammatory and oxidative stress pathways. Previous research has demonstrated that IR stimulates tumor growth indirectly by activating pro-inflammatory signaling pathways such as NF-κB [18]. Simultaneously, high triglyceride levels promote carcinogenesis by increasing the incidence of oxidative stress and the generation of reactive oxygen clusters [19]. It possesses the ability to directly affect the pancreas and raise the body’s level of oxygen free radicals, which can damage cellular DNA and cause tumors [20]. Increased lipid metabolism from elevated body lipids leads to increased adipocyte metabolism [21]. Adipocytes provide a protumorigenic environment and release inflammatory cytokines [22, 23]. Periprostatic adipocytes also encourage the extracapsular growth of tumor cells by secreting chemokines, which has an impact on the progression of cancer.

The anaerobic conversion of pyruvate to lactate is catalyzed by LDH, a ubiquitous cellular enzyme that serves as the rate-limiting step in this process [24]. Elevated serum LDH levels have been established as a prognostic biomarker in multiple malignancies, including lung, colorectal, and nasopharyngeal carcinomas [25]. Elevated serum LDH levels initiate a downstream signaling cascade mediated by HIF-1, leading to increased VEGF secretion by tumor cells. This process establishes a self-reinforcing cycle: heightened HIF-1 activity not only upregulates LDH expression but also creates a pro-angiogenic tumor microenvironment that facilitates tumor angiogenesis and metastatic progression [26, 27]. Furthermore, in line with the Warburg effect, plasma LDH levels show a positive correlation with critical tumor characteristics, including glucose uptake, metabolic activity, and invasiveness [28]. As a result, elevated serum LDH levels are strongly associated with poor clinical outcomes.

Several studies have demonstrated that an elevated TyG index is associated with an increased risk of PCa development [29]. Further supporting this, a large-scale European cohort study identified a significant correlation between the TyG index and PCa-specific mortality [30]. Additionally, serum LDH has emerged as a potential predictive biomarker with therapeutic relevance in PCa, as it has been consistently linked to adverse oncologic outcomes [31]. Beyond these findings, our study provides novel evidence that the TyG index and LDH levels may serve as accurate predictors of rapid disease progression in advanced HSPC.

Above all, with the aid of multivariate Cox regression analysis, a novel HSPC prognostic nomogram integrating TyG index, LDH, M stage and Gleason sum was established, and suggested more favorable discriminative and predictive ability compared with the M stage and Gleason sum, which were identified by ROC curve and DCA analysis. After dividing the patients into groups with low-, intermediate- and high-risk score, the group with a high-risk score developed rapidly and had a bad prognosis. Thus, the nomogram based risk system could provide a convenient and intuitive tool to initially classify advance HSPC patients into different prognostic stage. For instance, patients classified as high-risk HSPC are more likely to benefit from treatment intensification, such as doublet therapy combining ADT with either chemotherapy or androgen receptor signaling inhibitors (ARSI), or triplet therapy comprising ADT, ARSI, and chemotherapy. Notably, a significant proportion of Asian patients exhibit long-term responses to ADT or complete androgen blockade (CAB) [32], suggesting that upfront ARSI administration in all HSPC patients may constitute overtreatment. Conversely, low-risk HSPC patients often achieve favorable outcomes and may be suitable candidates for treatment de-escalation. Furthermore, by incorporating predicted PFS into clinical decision-making, tailored and timely follow-up strategies can be implemented to optimize patient management.

This study has several limitations. First, as a single-center investigation with a relatively small sample size, its generalizability may be limited. Second, while ADT combined with bicalutamide or flutamide is being progressively replaced by ARSI therapy in clinical practice, our findings from an Asian population may not be directly applicable to other ethnic groups due to potential differences in treatment responses and disease biology. Future studies should incorporate detailed treatment data to better elucidate the role of metabolic interventions in HSPC management. Additionally, multi-center, large-scale studies are needed to validate the associations between the TyG index, LDH levels, and clinical outcomes in HSPC patients.

In conclusion, the successful construction of a novel nomogram for HSPC patients based on M stage, Gleason sum, TyG index and LDH has revealed fresh knowledge, which will assist clinicians in quickly and accurately assessing the prognosis of patients and identifying high-risk HSPC in order to develop individualized treatment plans and follow-up strategies.

## Data Availability

The source code and data analyzed during the current study are available from the corresponding author on reasonable request.
